# Five metastasis-related mRNAs signature predicting the survival of patients with liver hepatocellular carcinoma

**DOI:** 10.1186/s12885-021-08431-1

**Published:** 2021-06-11

**Authors:** Chao Chen, Yan Qun Liu, Shi Xiang Qiu, Ya Li, Ning Jun Yu, Kang Liu, Li Ming Zhong

**Affiliations:** 1 North Sichuan Medical College, School of Medical Imaging, Nanchong, 637000 Sichuan China; 2 Nanchong Central Hospital,The Second Clinical Medical College of North Sichuan Medical College, Institute of Tissue Engineering and Stem Cell Research, Nanchong, 637000 Sichuan China; 3 Department of Interventional Radiology, The Second Clinical College of North Sichuan Medical College, Nan Chong Central Hospital, Nan Chong, 637000 Sichuan China

**Keywords:** Liver hepatocellular carcinoma, Metastasis, Prognostic model, TCGA, GSEA

## Abstract

**Backgrounds:**

Liver hepatocellular carcinoma (HCC) is one of the most malignant tumors, of which prognosis is unsatisfactory in most cases and metastatic of HCC often results in poor prognosis. In this study, we aimed to construct a metastasis- related mRNAs prognostic model to increase the accuracy of prediction of HCC prognosis.

**Methods:**

Three hundred seventy-four HCC samples and 50 normal samples were downloaded from The Cancer Genome Atlas (TCGA) database, involving transcriptomic and clinical data. Metastatic-related genes were acquired from HCMBD website at the same time. Two hundred thirty-three samples were randomly divided into train dataset and test dataset with a proportion of 1:1 by using caret package in R. Kaplan-Meier method and univariate Cox regression analysis and lasso regression analysis were performed to obtain metastasis-related mRNAs which played significant roles in prognosis. Then, using multivariate Cox regression analysis, a prognostic prediction model was established. Transcriptome and clinical data were combined to construct a prognostic model and a nomogram for OS evaluation. Functional enrichment in high- and low-risk groups were also analyzed by GSEA. An entire set based on The International Cancer Genome Consortium(ICGC) database was also applied to verify the model. The expression levels of SLC2A1, CDCA8, ATG10 and HOXD9 are higher in tumor samples and lower in normal tissue samples. The expression of TPM1 in clinical sample tissues is just the opposite.

**Results:**

One thousand eight hundred ninety-five metastasis-related mRNAs were screened and 6 mRNAs were associated with prognosis. The overall survival (OS)-related prognostic model based on 5 MRGs (TPM1,SLC2A1, CDCA8, ATG10 and HOXD9) was significantly stratified HCC patients into high- and low-risk groups. The AUC values of the 5-gene prognostic signature at 1 year, 2 years, and 3 years were 0.786,0.786 and 0.777. A risk score based on the signature was a significantly independent prognostic factor (HR = 1.434; 95%CI = 1.275–1.612; *P* < 0.001) for HCC patients. A nomogram which incorporated the 5-gene signature and clinical features was also built for prognostic prediction. GSEA results that low- and high-risk group had an obviously difference in part of pathways. The value of this model was validated in test dataset and ICGC database.

**Conclusion:**

Metastasis-related mRNAs prognostic model was verified that it had a predictable value on the prognosis of HCC, which could be helpful for gene targeted therapy.

## Introduction

Liver hepatocellular carcinoma had been the sixth most commonly diagnosed cancer and the second leading cause of cancer death worldwide in 2020, with about 905,677 new cases and 830,180 deaths annually [1]. The 5-year survival and Overall Survival rates are below 12%. Precursors of most HCC cases include liver cirrhosis, chronic hepatitis viral infections, alcohol-related liver disease, non-alcoholic fatty liver disease, and drug-induced hepatitis [2]. Curing HCC had been a complex issue for doctors since its birth. Surgery treatment was the main method of liver hepatocellular carcinoma. Interventional therapy is a way for patients which plays an important role in the therapy of advanced liver cancer, through a minimally invasive surgery to suppress its proliferation. However, prognosis was often not very good. HCC is a highly aggressive and heterogeneous disease [3]. For advanced HCC cases, moreover, the recurrence rate is nearly 80% with the patients and the metastasis rate is nearly 30%, whose 5-year survival rate is only 25–39% [4]. In addition, HCC’ metastasis and recurrence led to shorter survival time and worse survival quality. Metastasis of HCC is one of the important reasons for poor prognosis. Liver hepatocellular carcinoma usually metastasizes in liver in the early time and it is easy to invade portal vein and branches and form tumor thrombus, which will cause multiple metastases in the liver after falling off. Lung is the most common organ liver hepatocellular carcinoma metastasizes through blood. Hilar lymph nodes are the most common metastatic lymph nodes. It is because of the lack of symptoms and metastasizing in the liver in early stage that most patients lost opportunities for surgeries [5]. At the time of HCC diagnosis, only 5 to 15% of cases have the extrahepatic spread [6]. As a result, a demand for new markers to diagnose HCC and predict prognosis is of great urgency.

The metastatic establishment of cancers at distant organs is largely uncurable and primarily contributes to the deaths of cancer patients [7]. Many mRNAs had been reported that they were related to the metastasis of tumors or chemoresistance. For example, the overexpression of ACTN2 in human liver cancer cells enhanced cellular motility and invasion abilities which suggested it could be functional in liver cancers’ metabolism [8]. Misawa’s study showed that prostate cancer *HOXA11-AS* and HOXB13(a kind of metastasis gene) promote metastasis through CCL2/CCR2 signaling pathway in autocrine and paracrine manners [9]. HMGB1 was found that its suppression could be useful for CDDP sensitization [10]. In one hand, the abnormal of metastasis-related mRNAs had deeper relation to the metabolism and proliferation which could contribute to the prediction of tumors’ development. In other hand, Cancer lethality is mainly caused by metastasis. Therefore, understanding the nature of the mRNAs involved in this process has become a priority [11].

We designed a study which extracting a series of metastasis-related mRNAs and combining them with clinical data to find out whether they had connections with OS. This study was aimed to find a better way to evaluate HCC prognosis through analyzing HCC risk score system and establishing metastasis-related lncRNA prognostic model and guide clinical treatment.

## Methods

### Data collection and processing

**Fig. 1:**
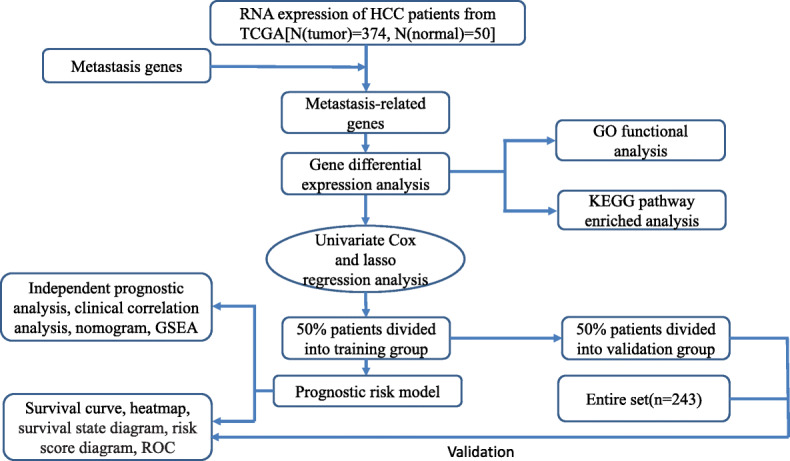
Overall study progress

**Fig. 2:**
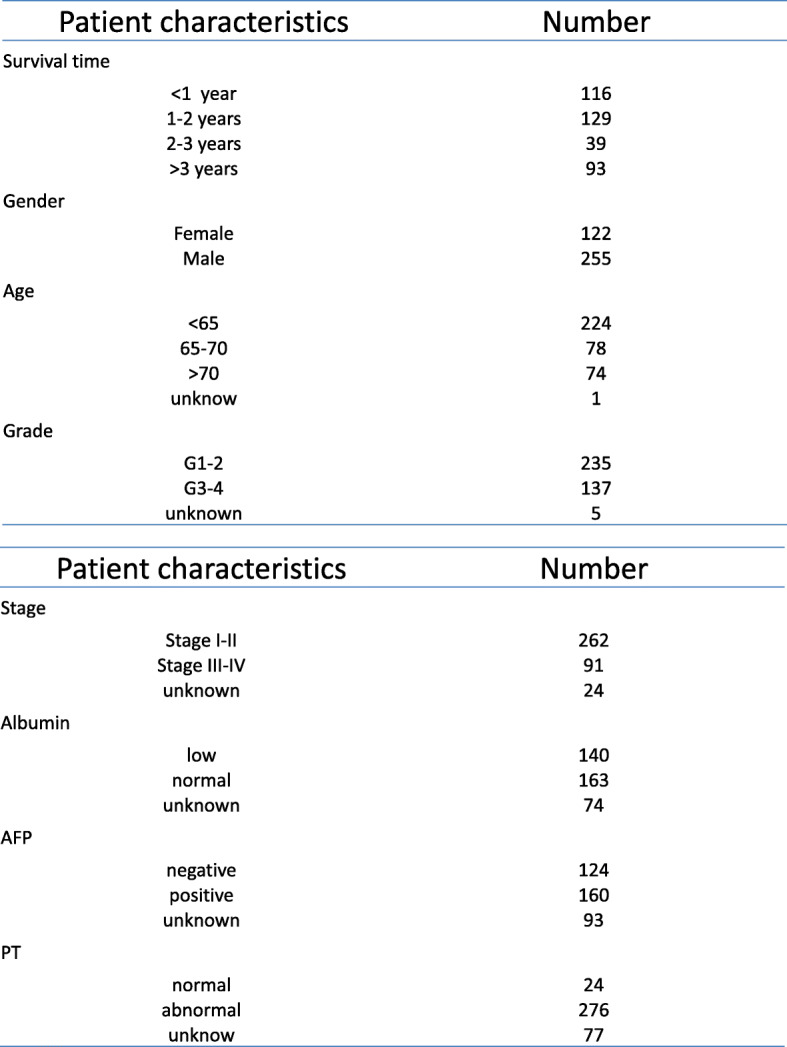
Clinical characteristics of 374 HCC patients

The overall study progress was showed in Fig. 1. Patients’ transcriptome and clinical data were downloaded from TCGA database. The former includes 374 cases of HCC and 50 normal cases and the latter involved age, gender, grade, stage, Alb, AFP, PT, Bilirubin and survival time, etc. The clinical characteristics of the 374 HCC patients was listed (Fig. 2.).The samples whose survival time was less than 30 days were deleted. We downloaded metastasis-related genes from HCBMD website and combined mRNAs which were expressed in HCC patients and metastasis-related genes. “limma” packages in R was used to distinguish the mRNAs which were expressed differently in tumor and normal samples. FDR less than 0.05 and |log2(FC)| higher than 1 were set as value. Then, 6 metastasis-related mRNAs had we obtained after we adopted univariate Cox regression analysis and lasso regression analysis to filter mRNAs. The gene which *p*-value was < 0.05 in univariate regression analysis was regarded as a candidate gene for prognosis. The HCC data sets of 233 TCGA patients used for prognosis analysis were divided into training set (*n* = 117) and invalidation set (*n* = 116) according to the proportion of 50 and 50% by using caret package in R software.

### Gene functional annotation of DE-MRGs

The Gene Ontology (GO) function [12] and Kyoto Encyclopedia of Genes and Genomes (KEGG) [13] pathway enrichment regarding the differentially expressed IRGs were analyzed using “ggplot2” and “ClusterProfiler” packages. The results which *p* < 0.05 were considered as statistically significant [12].

### Construction of regulatory network and prognostic model

In training dataset, only mRNAs with *P* < 0.05 according to multivariate Cox regression analysis were considered as prognostic metastasis-related mRNAs. After deleting the patients without complete clinical information, we calculated each patient’s risk score through constructing a prognostic model to evaluate HCC patients’ prognosis.

$$ \mathrm{Risk}\ \mathrm{Score}\left(\mathrm{patient}\right)={\Sigma}_{\mathrm{i}}\mathrm{Coefficient}\left(\mathrm{mRNAi}\right)\times \mathrm{Expression}\left(\mathrm{mRNAi}\right) $$

Patients and their mRNAs’ expression were combined and divided into two group according to risk scores. Data was classified by the median risk score threshold as high or low risk group. Also, mRNAs were divided into high and low expression group by the median expression threshold. Using the “survival” software package in R, the survival curve of different expression of metastasis-related mRNAs and different risk groups were visualized. In addition, we constructed a risk curve with package “survival” and “heatmap” in R. After that, a time-dependent ROC (receiver operating characteristic) curve for OS prediction and a 3-year time-dependent ROC curve of risk score and other clinical characteristics were used to assess the sensitivity and specificity of the prognosis through utilizing package “timeROC” in R.

### Clinical correlation analysis and validation of the prognostic model

Univariate and multivariate were implemented to confirm the independency of the prognostic model with or without clinical elements (age, gender, grade, stage, Alb, AFP, PT, Bilirubin). Thereafter, clinical characteristics and risk score were classified into two categories to protracted a nomogram. Calibration curves were made to exam the nomogram. Nomogram is widely used to predict cancer prognosis [14]. Since the model had been constructed, validation group and ICGC database (*n* = 232) were used to evaluate the feasibility of the model. Similarly, methods mentioned before were used in both testing cohorts. And the immunohistochemistry staining of both the normal and HCC samples were downloaded from the Human Protein Atlas database (https://www.proteinatlas.org/).

### Functional analysis

To reveal the KEGG pathways involved in high-risk and low-risk groups, GSEA analysis was performed to define the biological processes enriched in the gene rank between the two groups. GESA with Java program was implemented to analyze the functions of the prognostic model. The random sample permutation number was set as 1000, and the significance threshold was *P* < 0.05, adopting high risk versus low risk.

### Statistical analysis

All statistical analyses were performed using the R 4.0.3 software and Perl packages was utilized to arrange the data. Of all the process, *P* < 0.05 was recognized as statistically significant.

## Results

### GO and KEGG functional annotations

**Fig. 3:**
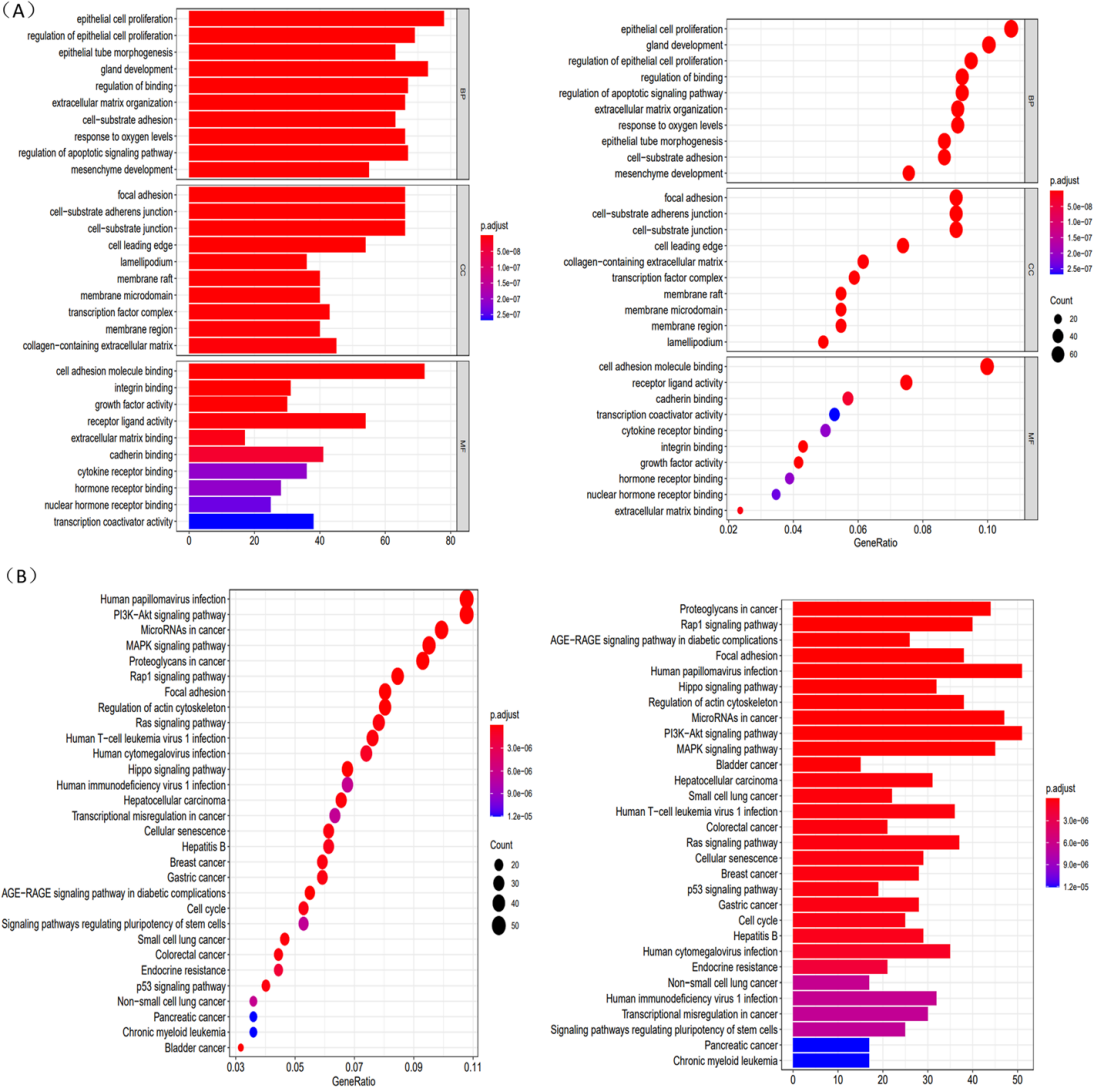
Gene functional enrichment of differentially expressed IRGs. **A** Gene ontology analysis; **B** The Kyoto Encyclopedia of Genes and Genomes analysis

GO analysis demonstrated that these DEGs were significantly enriched in biological process (BP), including epithelial cell proliferation, gland development, regulation of epithelial cell proliferation, regulation of blinding and regulation of apoptotic signaling pathway. In addition, in the cellular component (CC) analysis, these DE-MRGs were enriched in the focal adhesion, focal adhesion and cell−substrate junction. It was suggested that in molecular function (MF), cell adhesion molecule binding and receptor ligand activity were enriched mostly. (Fig. 3A). KEGG analysis results showed that these mRNAs mainly enriched in Human papillomavirus infection and PI3K − Akt signaling pathway (Fig. 3B).

### Metastasis-related mRNAs prognostic model and survival analysis

**Fig. 4:**
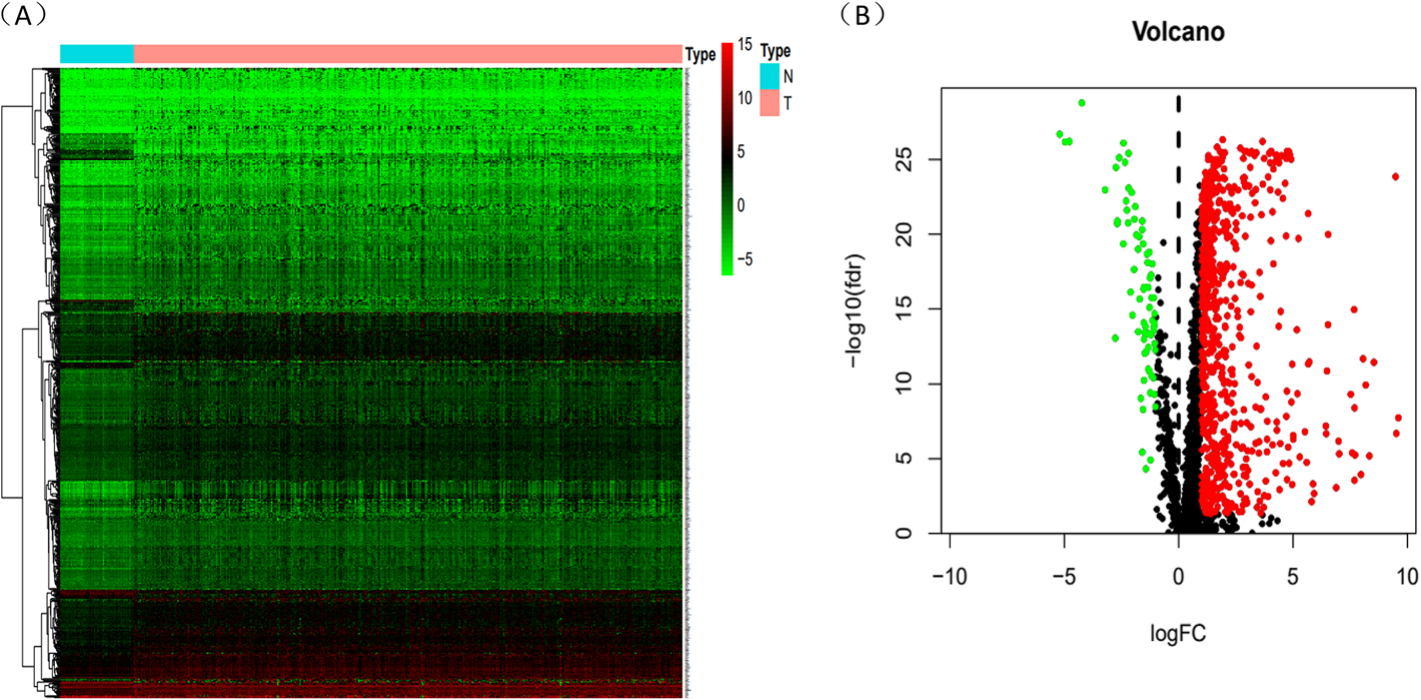
Identification of differentially expressed metastasis-related mRNAs. **A**-**B** Heatmap and volcano plot of differentially expressed mRNAs in HCC based on data from TCGA

**Fig. 5:**
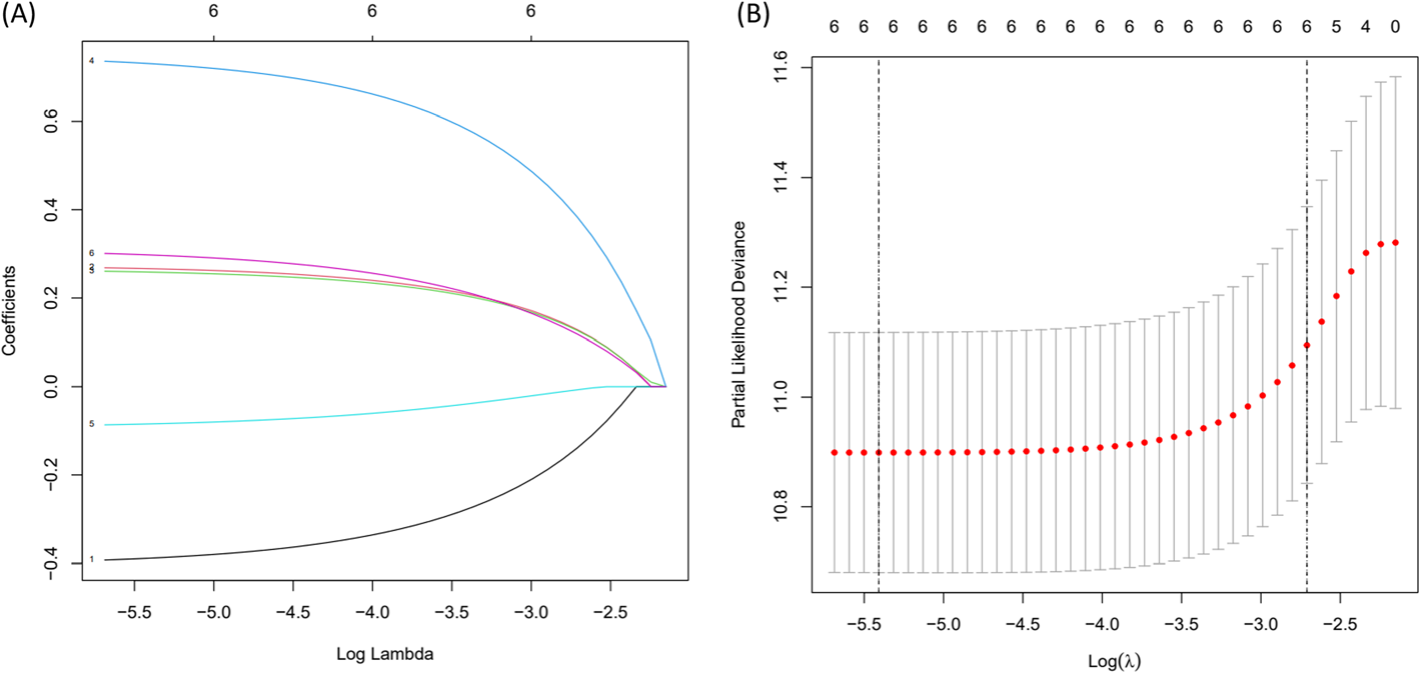
**A**-**B** The coefficients calculated by LASSO

One thousand eight hundred ninety-five mRNAs were screened after metastasis-related genes and mRNAs which were expressed in HCC patients were overlapped. By intersecting the DEGs and metastasis-related mRNAs in HCC, 666 mRNAs were up-regulated and 73 mRNAs were down-regulated among 739 different expression mRNAs. The DEGs were showed in a heatmap and a volcano plot (Fig. 4A,B). Whereafter, through univariate Cox regression analysis with *p* < 0.05, we extracted 6 metastasis-related mRNAs associated with prognosis. Next, lasso regression analysis (Fig. 5A,B) and multivariate Cox regression analysis were performed to screen the candidate mRNAs. As a result, we acquired 5 MRGs (TPM1,SLC2A1, CDCA8, ATG10 and HOXD9) which could be used to construct prognostic model. SLC2A1, CDCA8, ATG10 and HOXD9 were considered as negative factors while TPM1 was considerd as postive factor. Multivariate Cox regression analysis was performed to establish prognostic model and construct a risk score prognostic index. Risk score was calculated by the following formula: [Expression level of SLC2A1 ∗ (0.33)] + [Expression level of ATG10 ∗ (0.8)] + [Expression level of HOXD9 ∗ (0.41)] + [Expression level of CDCA8 ∗ (0.55)] − [Expression level of TPM1 ∗ (0.37)].

**Fig. 6:**
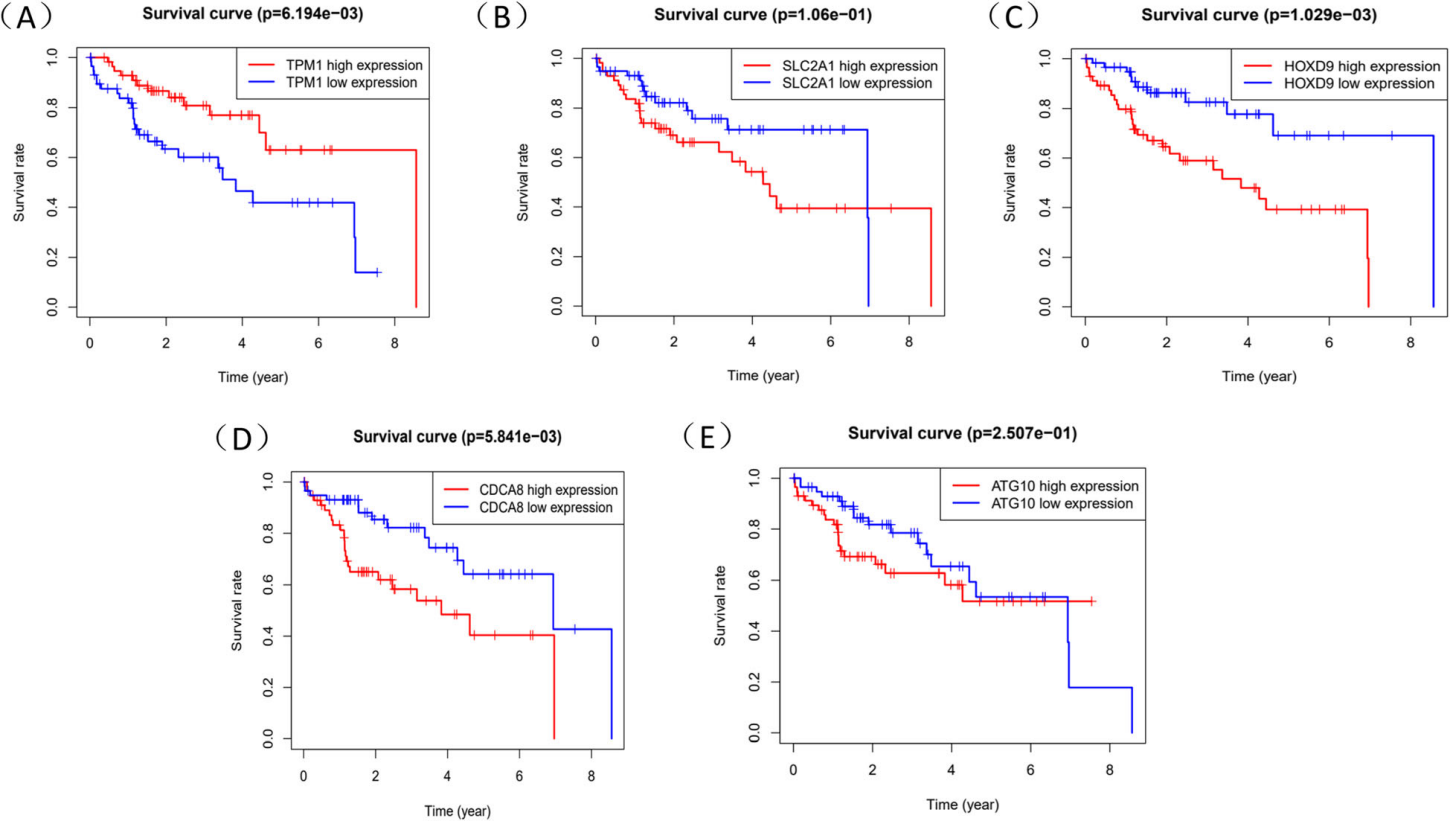
Prognostic gene survival curve. **A** TPM1 **B** SLC2A1. **C** HOXD9. **D** CDCA8. **E** ATG10

**Fig. 7:**
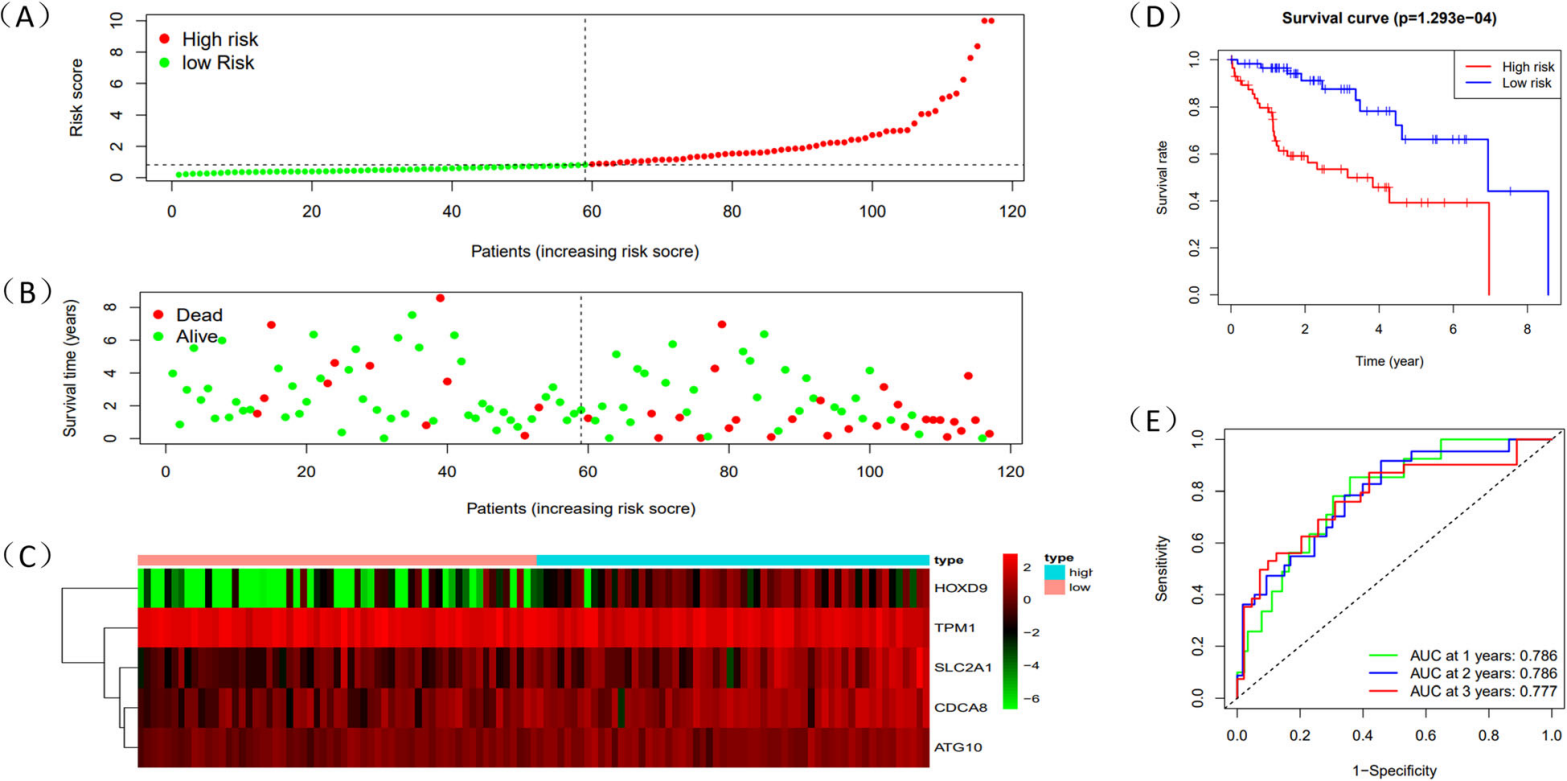
The prognostic results of risk score in train dataset. **A** Rank of prognostic index and distribution of high and low risk groups. **B** Survival status of patients in different groups. **C** Heatmap of expression profiles of included mRNAs in high and low risk groups. **D** Survival curves of the HCC patients with different risk scores. **E** Time-dependent ROC curves of the risk model for the 1-, 2- and 3-year survival

**Fig. 8:**
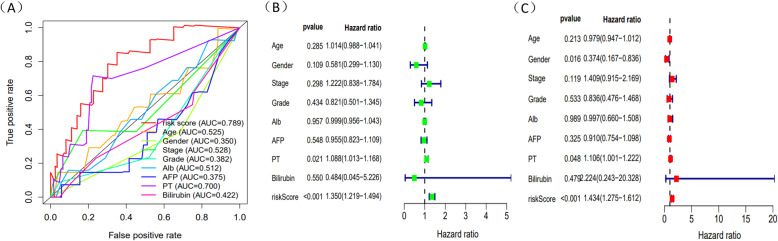
Cox analysis of the 5 genes signature. **A** The 3-year time-dependent ROC curve of risk score and other clinical characters in the HCC patients (AUC = 0.789). **B** Univariate Cox regression analysis of characteristics and risk score of the HCC patients in the train dataset. **C** Multivariate Cox regression analysis of characteristics and risk score of the HCC patients in the train dataset

Through the level of Risk score, HCC patients could be split into two groups: high-risk and low-risk group. Risk score which was higher than median would be classified as the high-risk group. Survival time of 5 mRNAs was displayed by “survival” package in R (Fig. 6A-E). Meanwhile, R was used to draw survival curve of 5 mRNAs according to different risk score (Fig. 7A-C). As is shown in the figures, with the growth of risk scores, the risk rating increases gradually. The same was true in the survival diagram and heatmap of 5 mRNAs. The survival intervals could be significantly differentiated in HCC patients with high and low risk. Survival analysis showed that high-risk group had shorter overall survival time than low-risk group (Fig. 7D). Such results showed that risk score could be a potential index for prognosis prediction of HCC patients. We also drew a ROC curve to assess the sensitivity and specificity of the model for 1-,2-,3-year survival, with an AUC of 0.786,0.786 and 0.777 (Fig. 7E). In addition, a 3-year time-dependent ROC curve of risk score and other clinical characteristics such as grade, stage, AFP etc. was made to compare the sensitivity and specificity between the risk score and others clinical charaters. Results showed that our model had a better sensitivity and specificity than others, with an AUC of 0.789 (Fig. 8A).

### Independent prognostic analysis and clinical correlation analysis

**Fig. 9:**
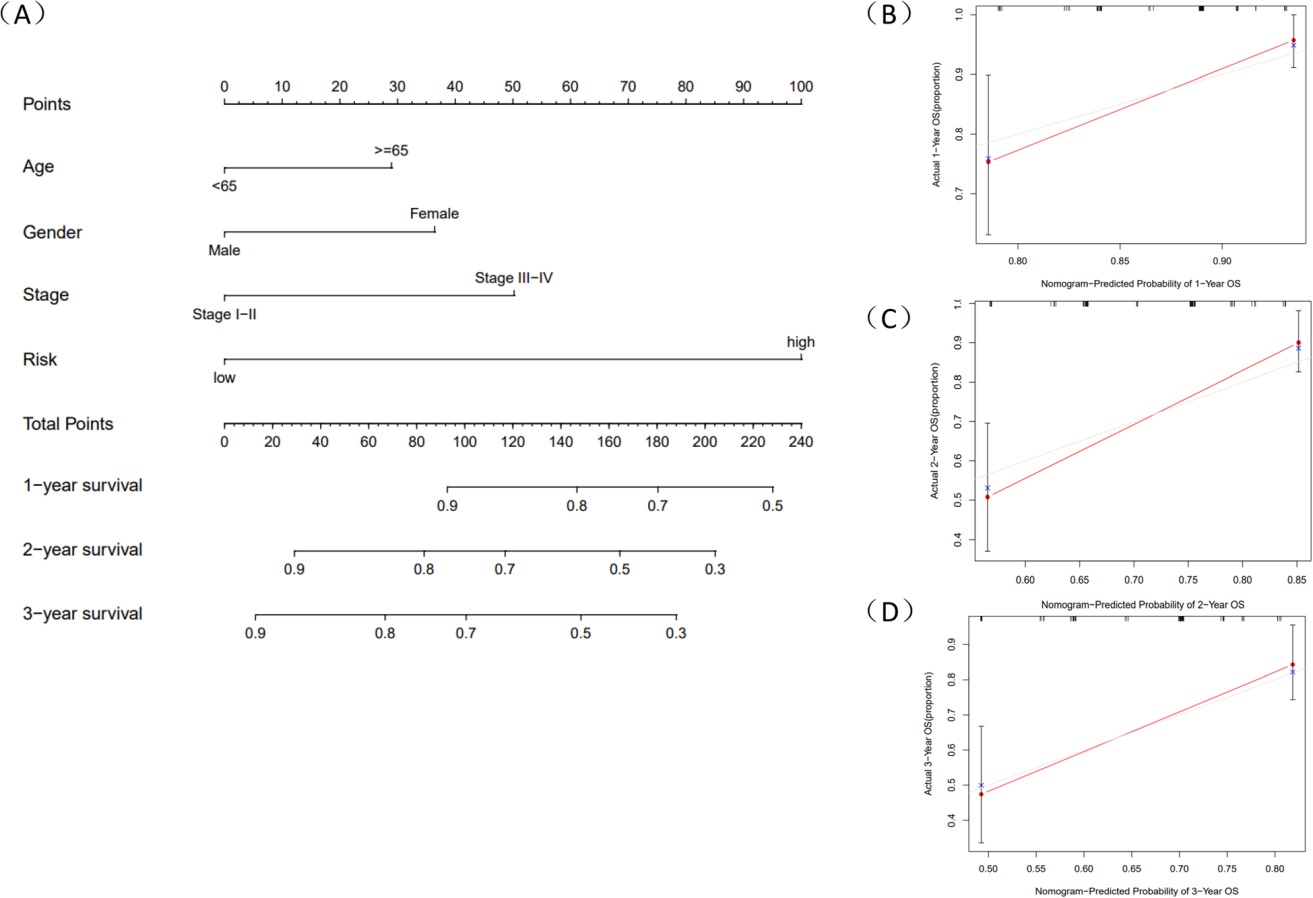
The nomogram to predict 1-,2, and 3-year OS and prognostic value of 5 mRNAs in the training cohort. **A** Nomogram for OS at 1-, 2- and 3-year in HCC patients. **B**-**D** Calibration plot at 1-, 2- and 3-year for validation to predict the probability of OS

**Fig. 10:**
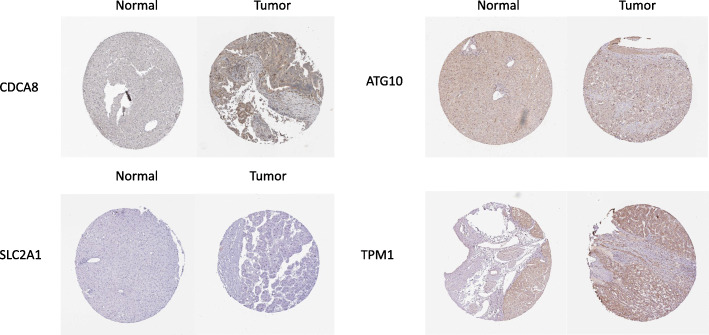
The protein expression of CDCA8,ATG10,SLC2A1 and TPM1 in liver cancer from the Human Protein Atlas

Since constructing a risk score model, we integrated the model with clinical characteristics and adopted univariate and multivariate Cox regression analysis to identify whether risk score could be an independent prognostic factor. Results showed that risk score could evaluate prognosis by itself in univariate Cox regression analysis (*P* < 0.05) (Fig. 8B) and it could also be an obviously predictable factor for prognosis after eliminating the influence of other characteristics **(**Fig. 8C). We used a method of bisection to divide the clinical traits (including age, gender, grade, stage) and risk score into two groups. Results indicated that with the growth of tumors’ stage, the risk score of patients increased (*P* < 0.05) and other characteristics didn’t show significant connection. Similarly, we employed package “rms” to draw an OS nomogram at 1-, 2- and 3-year in HCC patients. We performed subgroup analysis of the two signatures in age (< 65, > = 65), clinical stage (stage I-II, stage III-IV), gender (female, male) and riskScore (low, high). Results showed that shorter OS happened in subgroup of > = 65, stage III-IV and high-risk. In addition, a verified calibration curve for 1-, 2- and 3-year was plotted to testify the nomogram (Fig. 9A-D). Moreover, we validated the 5 genes using immunohistochemistry. The protein expression of TPM1,SLC2A1, CDCA8, ATG10 was also displayed according to the Human Protein Atlas(Fig. 10). In additional, according to the study of Zhu [15], HoXD9 was found that it promotes the proliferation and invasive capacity of GC cells However, the expression of HOXD9 in HCC was not found on the website. It is worth studying in detail in the future.

### Functional analysis of prognostic model

**Fig. 11:**
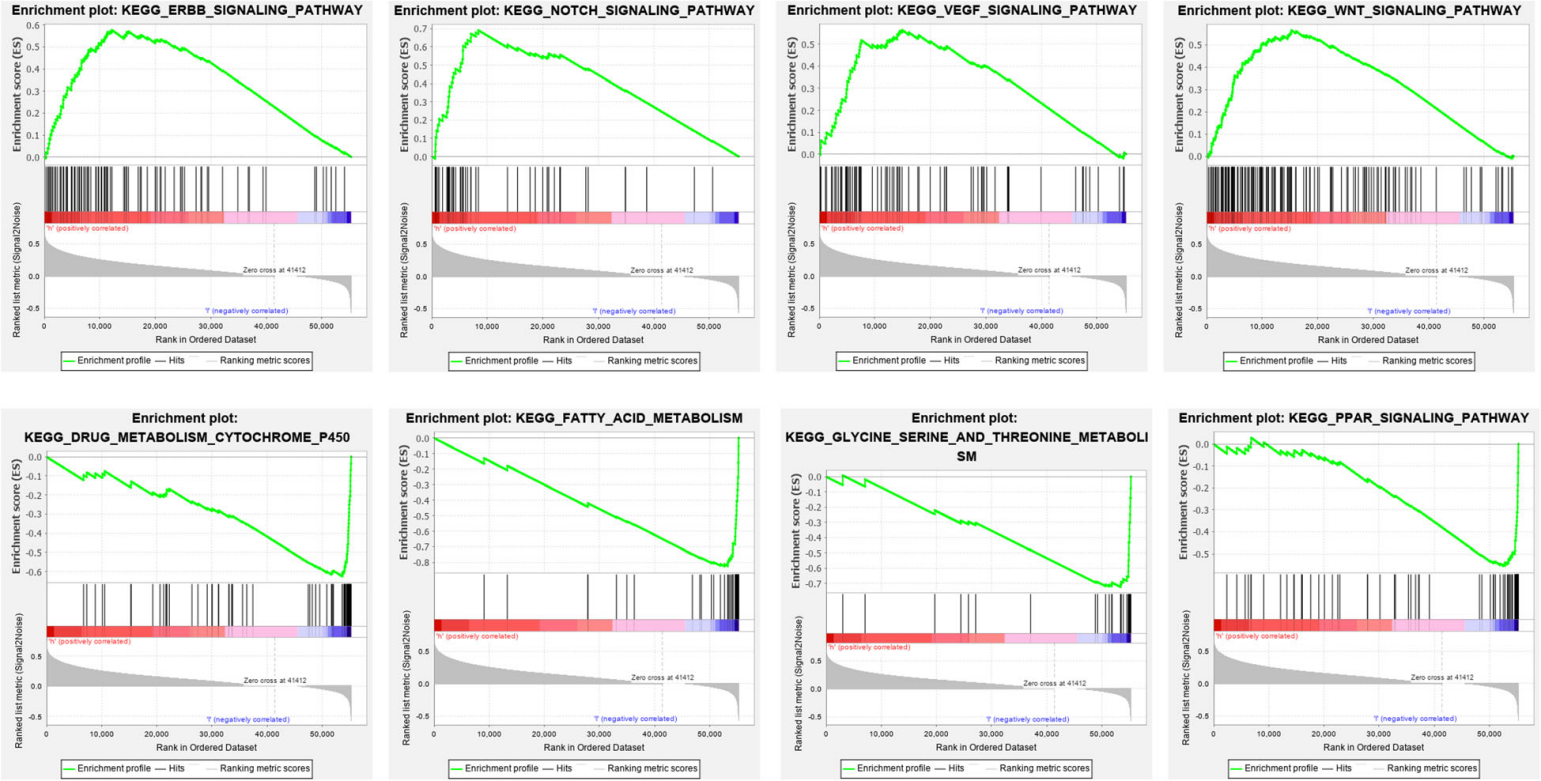
Gene Set Enrichment Analysis in TCGA database. Enrichment Map were used for visualization of the GSEA results

KEGG enriched analysis had we adopted to find out which pathway related to cancer would the mRNAs focus on and validate the biological function of the constructed model. Results (Fig. 11) showed that mRNAs which were of high score had an obvious enrichment in pathways such as “Notch signaling pathway”, “VEGF signaling pathway”, “WNT signaling pathway”, “ERBB signaling pathway” etc. while “PPAR signaling pathway”, “drug metabolism-cytochrome p450”, “fatty acid metabolism” and “Glycine serine and threonine metabolism” etc. were enriched in low-risk group.

### Validation of the model

**Fig. 12:**
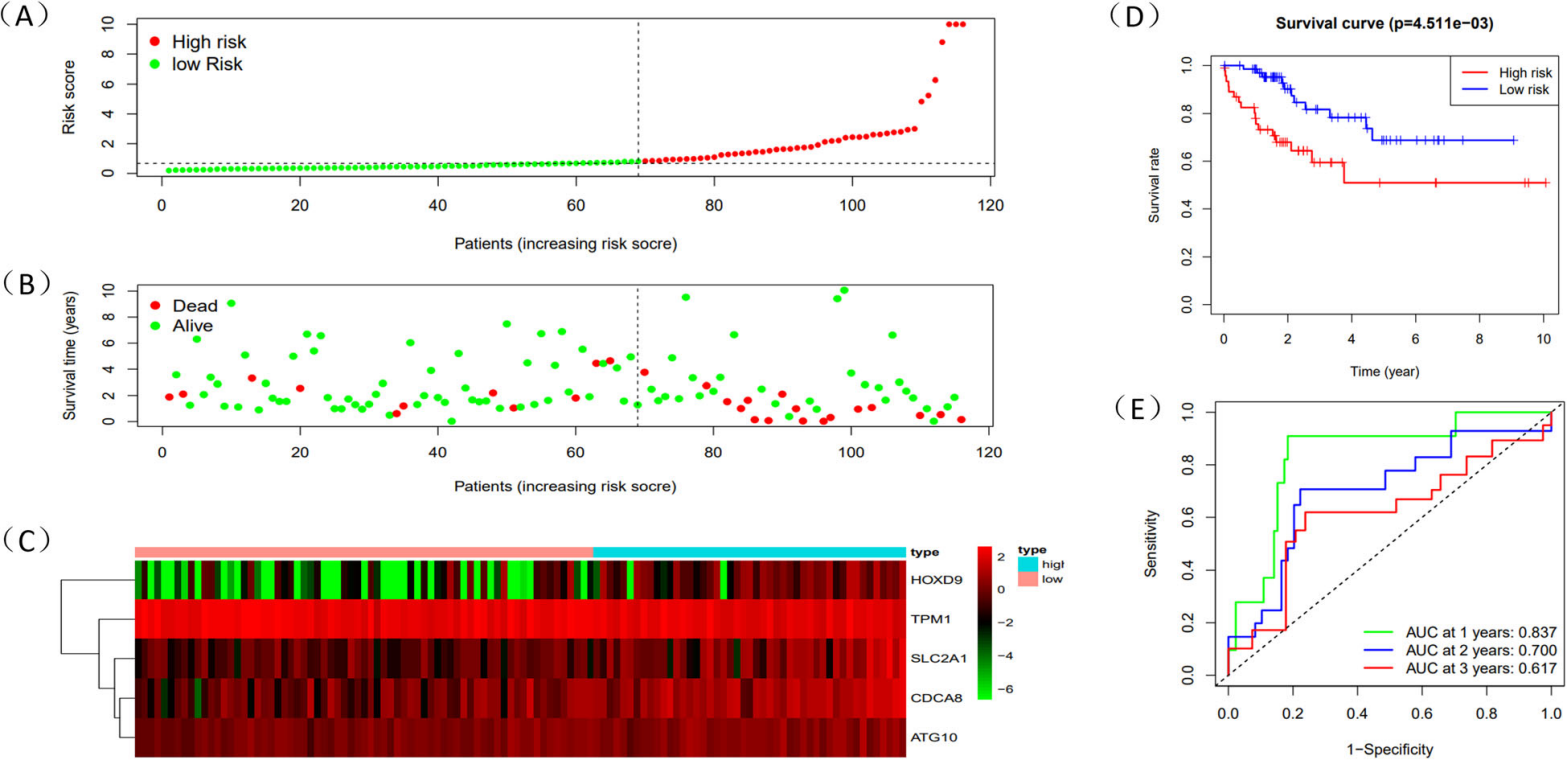
The prognostic results of risk score in TCGA testing dataset. **A** Rank of prognostic index and distribution of high and low risk groups. **B** Survival status of patients in different groups. **C** Heatmap of expression profiles of included mRNAs in high and low risk groups. **D** Survival curves of the HCC patients with different risk scores. **E** Time-dependent ROC curves of the risk model for the 1-, 2- and 3-year survival

**Fig. 13:**
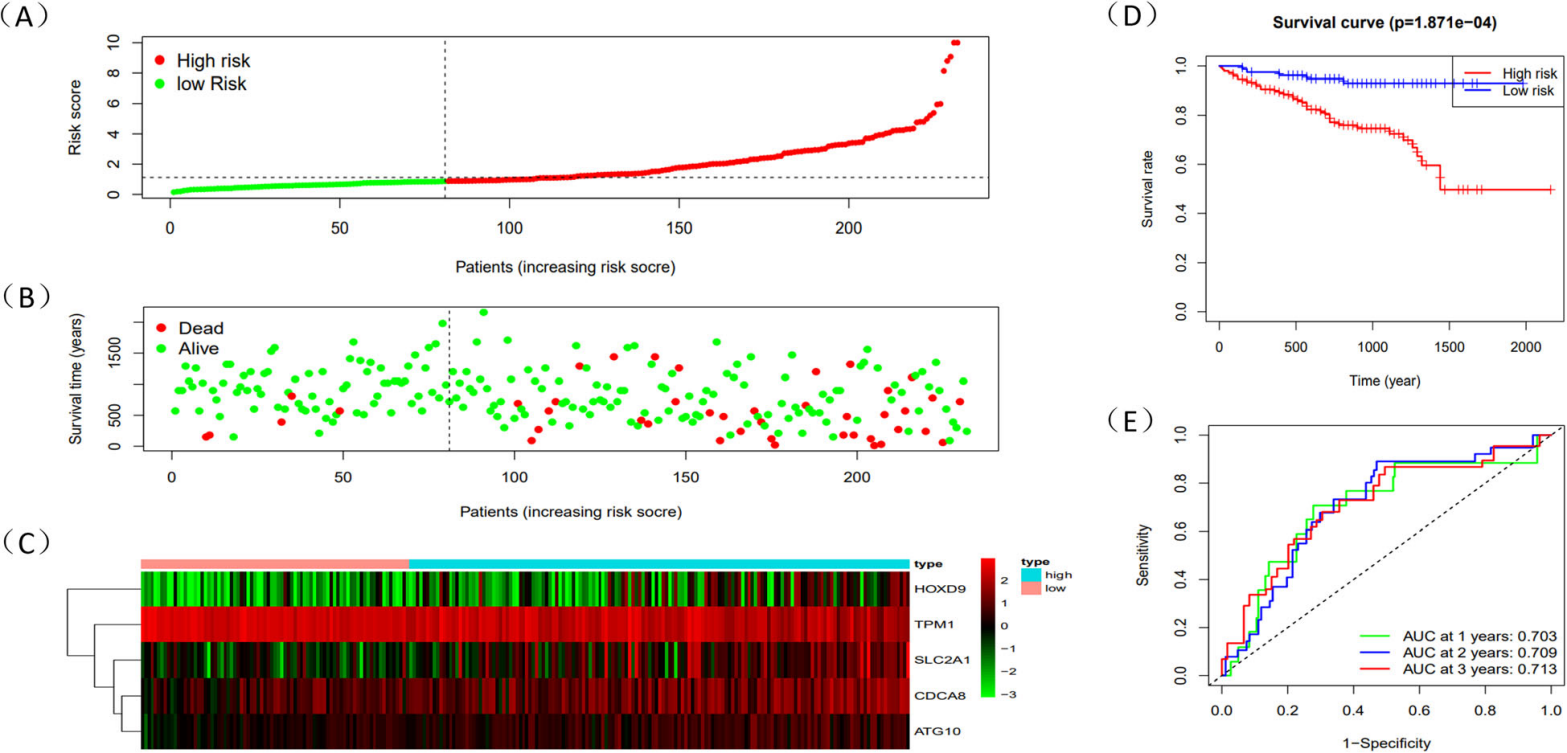
The prognostic results of risk score in the ICGC database. **A** Rank of prognostic index and distribution of high and low risk groups. **B** Survival status of patients in different groups. **C** Heatmap of expression profiles of included mRNAs in high and low risk groups. **D** Survival curves of the HCC patients with different risk scores. **E** Time-dependent ROC curves of the risk model for the 1-, 2- and 3-year survival

**Fig. 14:**
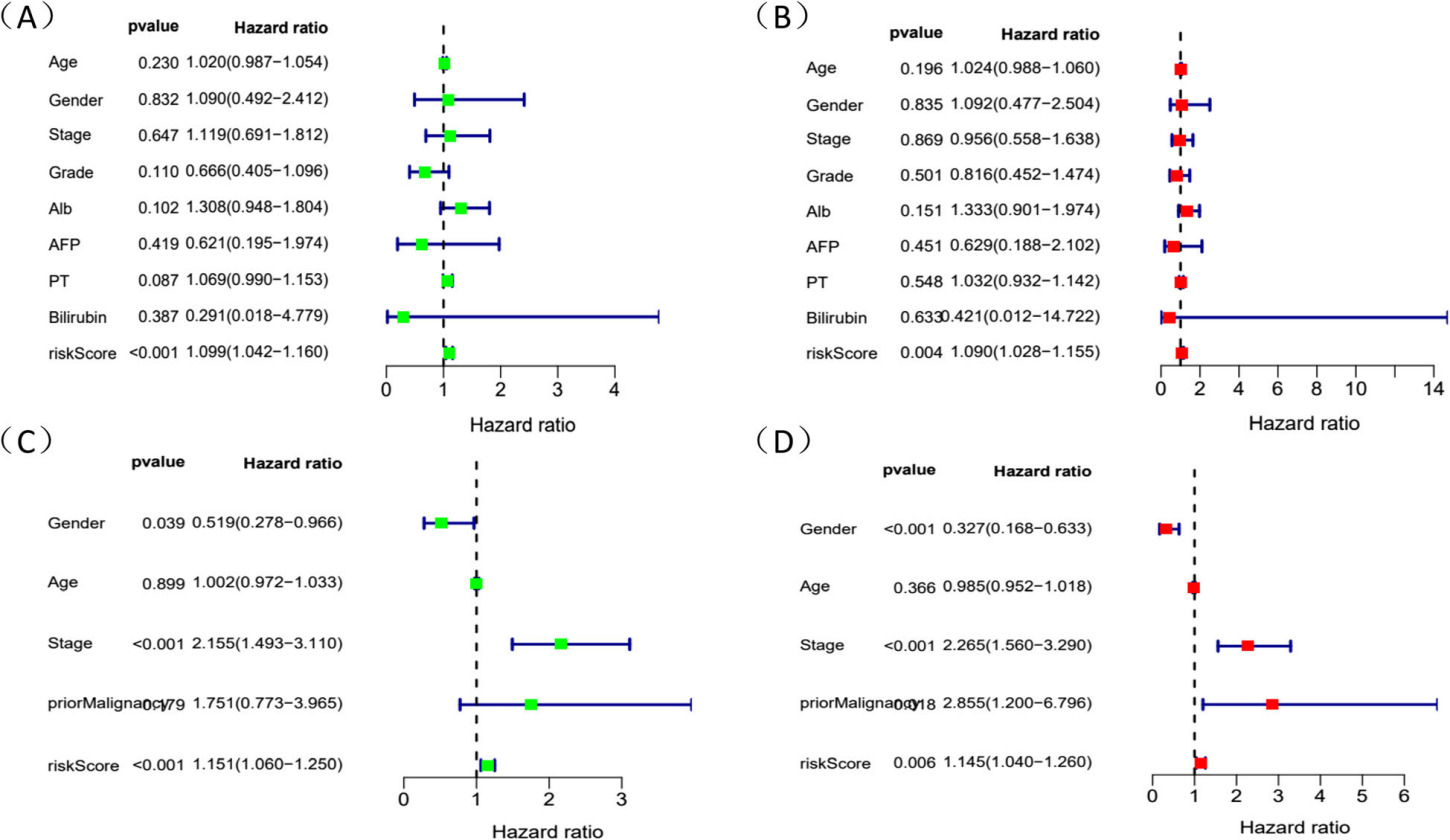
Cox analysis of the 5 mRNAs signature in 2 testing cohorts. **A**-**B** Unit and Multivariate Cox regression analysis of characteristics and risk score of the HCC patients in testing cohort. **C**-**D** Unit and Multivariate Cox regression analysis of characteristics and risk score of the HCC patients in the ICGC database

Following the primary methods and same coefficients, we established two risk score models coming from testing dataset to verify the accuracy of the metastasis-related prognosis model constructed before and all of the 5 metastasis-related mRNAs were validated in TCGA testing data and ICGC database. Patients from the testing cohort were also divided into high or low risk group. Survival curve was supplemented in both two groups (Figs. 12A-C and 13A-C). In line with the results of the TCGA testing cohort and ICGC database, patients who were classified as high-risk also had significantly inferior OS than the low-risk group (*P* < 0.05) (Figs. 12D and 13D). To assess the predictive performance of the 5-gene based signature, we constructed a time-dependent ROC curve in two validation groups (Figs. 12E and 13E), with an AUC of 0.837,0.700,0.617 for 1-,2-,3- year survival in testing cohort and an AUC of 0.703,0.709,0.713 for 1-,2-,3- year survival in ICGC database. Similarly, we found that risk score could be an independent factor through both univariate and multivariate Cox regression analysis (*P* < 0.05) (Fig. 14A-D).

## Discussion

Liver cancer has been one of the most diseases contributed to death and many prognostic models had been established to predict the prognosis of HCC. For example, autophagy-related long non-coding RNAs prognostic model [16], immune-related long non-coding RNAs prognostic model [17] and metastasis-related miRNAs [18] etc. Here we established a metastasis-related mRNAs prognostic model to predict the prognosis of HCC.

In this article, of all the analysis, HCC samples were randomly classified into training cohort, and testing cohort. Training cohort was used to construct a prognostic model, while testing cohort was utilized for validation. Firstly, we analyzed the gene expression data and clinical data of HCC patients enrolled in TCGA, discerning 881 mRNAs related to metastasis. Using univariate, lasso and multivariate Cox regression analysis, 5 mRNAs (TPM1,SLC2A1, CDCA8, ATG10 and HOXD9) had we found were detected as independent prognosis predictors in HCC. Secondly, survival analysis was utilized to examine the availability of the prognostic model. The high expression of all the 5 mRNAs, had a positive correlation to OS which meant that with the generate of these mRNAs’ expression, patients would have a longer survival time. The results suggested that metastasis-related mRNAs model was a significant prognostic factor for HCC patients. Thirdly, the model constructed in training group was validated internally and externally, adding dependability to the outcomes.

The mRNAs mentioned before had been reported in other articles that they also had relationship with different types of cancers. A study from Wen et al. [19] found that in cervical cancer the high level of HOXD9 is closely linked to metastasis rate and poor prognosis in cervical cancer patients. The downregulation of TPM1 enhanced prostate cancer cell proliferation, invasion and migration via cell-derived exosomal miR-183 in prostate cancer [20]. Shen et al. [21] found that through silencing the expression of ATG10, the migration and invasion of thyroid carcinoma could be inhibited. Moreover, high expression of CDCA8 promoted the proliferation of ovarian cancer cells in vitro and in vivo which increased the tumorigenesis, aggressiveness and chemoresistance of ovarian cancer [22]. High SLC2A1 expression was associated with poor prognosis, cancer cell proliferation, decreased immune cells, including CD8 T cells and B cells which contributing to the death of gastric caner patients [23]. Those results had represented similar conclusions as this study.

GSEA enriched manifested these mRNAs had effects on prognosis probably via several signaling pathways. For example, ERBB signaling pathway was found that it was related to hepatocellular carcinoma [24] while the progress of hepatocellular carcinoma had a relationship with Notch signaling pathway through a series of target spots [25, 26]. Moreover, VEGF signaling pathway [27, 28], WNT signaling pathway [29, 30] and PPAR signaling pathway [31] suggested similar results. Such findings showed the biological functions of mRNAs could be regarded as predictable factor for prognosis though more researches and experiments needed to done to verify the hypothesis. These results help us to explore the mechanism of metastasis-related mRNAs.

In conclusion, through a series of bioinformation analysis, we constructed a model and set up a biomarker for predicting the prognosis of HCC. Our study showed that patients who were in low-risk group had better OS than those in high-risk group. Such results were verified in both train and test cohort. Hence, this model had a good sensitivity and specificity on 1 year-, 2 years-, 3 years- survival time for HCC patients, having AUC values of the models at 1 year-,2 years-,3 years- survival were varied from 0.777 to 0.786. This study was highly methodologically reasonable because we downloaded data from TCGA database which contained great amounts of samples and opened a new prospect for the regulation of metabolic processes and the treatment [32] of HCC. However, there are several deficiencies in this study. Firstly, data in TCGA may have variable degrees of errors and the amount of data included is not large, which may cause inaccuracy. Secondly, lacking of experiments in vivo and in vitro will lead to insufficient evidence for this model. Thus, future studies and more experiments should be implemented to validate the model and biomarker and ensure its robustness.

## Conclusion

We constructed a 5 metastasis-related mRNAs prognostic model based on MRGs and separated HCC patients from two groups. The survival outcomes of the two groups were statistically different, which meant that the disparate expression of MRGs may have effects on patients’ prognosis. These findings may promote the development of new biomarkers and targeted therapies. Therefore, the 5 metastasis-related mRNAs and might be molecular biomarkers and therapeutic targets for the patients with liver hepatocellular carcinoma.

## Data Availability

All data generated or analysed during this study are included in this published article.
